# Evaporation-assisted patterning beyond random assembly

**DOI:** 10.1093/nsr/nwz125

**Published:** 2019-09-03

**Authors:** Chuanhua Duan

**Affiliations:** Department of Mechanical Engineering, Boston University, USA

Evaporation of solvent and the resulting fluid motion has been recognized as a simple but robust patterning method to yield self-assembled patterns of non-volatile solutes (e.g. microspheres, nanoparticles, bacteria, polymers, proteins, DNA, graphenes, etc.) on plain surfaces [1,2]. Such evaporation-assisted patterning could occur at small scales (O(mm^2^)) by depositing and drying individual drops on surfaces, or at larger scales (O(cm^2^)) by continuously moving an evaporating meniscus.

**Figure 1.**
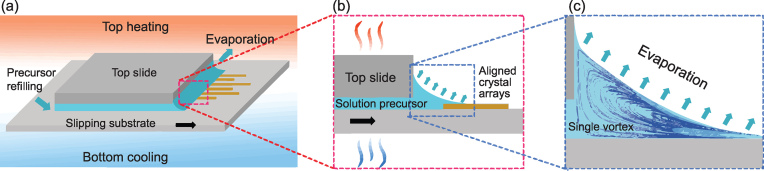
Schematics of evaporation-assisted patterning for material crystallization and alignment. (a) Schematic illustrations of the slipping-substrate set-up with the top-heating–bottom-cooling evaporation condition for large-scale patterns. (b) Zoomed-in cross-sectional view of the solution meniscus area where the aligned crystal arrays grow. (c) Zoomed-in cross-sectional view of the meniscus, showing a single Marangoni vortex. Adapted from [4].

While the resulting patterns have found promising applications in photonic crystals, biosensors, and anti-reflective coatings, they are usually randomly assembled and/or oriented because of the spatially varying evaporation flux at the liquid–vapor interface and the associated complicated fluid motion [1,2]. On the other hand, current evaporation-assisted patterning techniques only use evaporation to guide assembly of pre-synthesized materials, not synthesis of complex materials, e.g. crystal formation/growth of functional organic/inorganic crystals, which could also be significantly affected by fluid motion.

Clearly, the full potentials of evaporation-assisted patterning have not been exploited. Despite their simplicity and scalability, current evaporation-assisted patterning techniques are incapable of generating patterns of highly ordered and complex functional materials, which would have much wider applications than those offered by randomly assembled/oriented patterns, such as optoelectronics, integrated electronics, photovoltaics, and optical imaging [3].

How can we further improve evaporation-assisted pattering beyond random assembly? In a recent paper published in *National Science Review* [4], Li *et al.* provided a simple but elegant solution to this question, pushing evaporation-assisted patterning to an unprecedented new level. The authors used both experimental and simulation approaches to study evaporation-induced fluid flow and the resulting synthesis and assembly of iodide perovskite crystals in a wedge-shaped meniscus under different evaporation conditions. They found that normal ambient evaporation and bottom heating evaporation would result in multiple irregular Marangoni vortexes in the meniscus and generate crystal patterns with low quality and poor alignment. However, by using a top-heating–bottom-cooling (THBC) evaporation condition, evaporation would result in a single Marangoni vortex in the meniscus, which in turn would lead to formation of oriented high-quality crystal arrays.

By combining this THBC evaporation-assisted patterning strategy with a slipping substrate design (as schematically shown in Fig. 1), Li *et al.* successfully fabricated centimeter-scale thin films of dense and highly ordered iodide perovskite crystal patterns on different substrates and further confirmed the excellent optoelectronic properties of the resulting thin films for photovoltaic applications. They also used the THBC evaporation-assisted patterning for crystallization and assembly of other materials, including C_60_, 2,4,5-triphenylimidazole (TPI), 9,10-bis(phenylethynyl)anthracene (BPEA) and silver chloride nanowires. They demonstrated that, for each different solvent and substrate, there is a special window (in terms of top heat flux and bottom temperature) to guarantee aligned crystallization and/or assembly.

Li *et al.*’s work showed that THBC evaporation-assisted patterning is a general approach to prepare ordered patterns of functional materials. This approach can be applied to the same substrate multiple times to prepare thin films of hybrid materials. It can be further combined with other controlling processes, e.g. programming the moving rate and direction of bottom substrate or using top slide (or bottom substrate) with pre-designed micro/nanostructures [1], to directly create desired micro/nanoscale patterns of functional materials without using any lithography processes. This potential, together with its simplicity, scalability, low cost and short processing time, will make THBC evaporation-assisted patterning a very valuable advancement for thin-film formation and patterning.
